# Effect of Direct-Acting Antiviral Agents on Gastroesophageal Varices in Patients with Hepatitis C Virus-Related Cirrhosis

**DOI:** 10.3390/medicina58081077

**Published:** 2022-08-10

**Authors:** Hiroshi Hisanaga, Hidetoshi Takedatsu, Keigo Emori, Hiroto Inoue, Yasuhumi Kunitake, Tomoyuki Nakane, Shuhei Fukunaga, Tatsuya Ide, Keiichi Mitsuyama, Takuji Torimura

**Affiliations:** Department of Medicine, Division of Gastroenterology, Kurume University School of Medicine, Kurume 830-0011, Fukuoka, Japan

**Keywords:** antiviral agents, liver cirrhosis, esophageal and gastric varices

## Abstract

*Aim:* In patients with hepatitis C virus-related liver cirrhosis (LC) who achieve sustained virological responses (SVRs) through treatment with direct-acting antiviral agents (DAAs), it remains unclear whether there are improvements in gastroesophageal varices (GEVs) and portal hypertension. We investigated changes in liver function and GEVs that occurred after DAA therapy. *Materials and Methods*: We evaluated the medical records of 195 patients with hepatitis C virus-related LC who received DAAs. A total of 171 patients achieved SVRs, among whom 36 had GEVs before or after receiving DAA therapy. The liver function, fibrosis, and GEVs were re-evaluated every 6 months after receiving DAA therapy. The risk factors for progressive GEVs were investigated. *Results*: DAA therapy resulted in improvements in liver function (indicated by aspartate transaminase, alanine transaminase, and serum albumin levels) and fibrosis (indicated by type IV collagen levels and the Fibrosis-4 index). After receiving DAA therapy, 27 patients had stable GEVs and 9 had progressive GEVs. With respect to GEV grades before DAA therapy, there was a significant difference between patients with stable and progressive GEVs (*p* = 0.027). Presence of grade-2 GEVs before starting DAA therapy was a risk factor for GEV progression (odds ratio: 5.83; *p* = 0.04). Patients with grade-2 GEVs had significantly shorter progression-free periods than those with grade < 2 GEVs (*p* = 0.025). *Conclusions*: DAA therapy does not ameliorate GEVs. Furthermore, grade-2 GEVs can worsen after DAA therapy. Therefore, patients with GEVs of grades ≥ 2 should undergo endoscopic surveillance after receiving DAAs.

## 1. Introduction

Chronic hepatitis C virus (HCV) infection causes complications such as liver cirrhosis (LC), portal hypertension (PH), and hepatocellular carcinoma (HCC); moreover, this condition influences morbidity and mortality worldwide [1,2]. As PH progresses, additional complications that further aggravate LC, such as ascites, hepatic encephalopathy, and bleeding from gastroesophageal varices (GEVs), develop [3]; these complications contribute substantially to morbidity and mortality [4,5]. Since the risk of clinical decompensation and mortality in patients with LC who do not have GEVs is less than that in patients with LC who have GEVs [6], patients with LC need to be screened for the presence of GEVs [7,8].

The advent of the use of direct-acting antiviral agents (DAAs) has led to a significant improvement in HCV eradication, and most patients with HCV infection can achieve a sustained virological response (SVR) [9]. Furthermore, through treatment with DAAs, patients with LC, including those with decompensated cirrhosis, can also achieve SVRs. However, to the best of our knowledge, the long-term clinical benefits of DAA-induced SVRs have not been adequately evaluated [10]; therefore, it is important to perform such evaluations.

Afdhal et al. [11] demonstrated that patients with chronic HCV infection and compensated or decompensated cirrhosis who achieve SVRs can have clinically meaningful reductions in the hepatic venous pressure gradient (HVPG) even after long follow-up periods. It was found in another study that PH is ameliorated by DAA-induced SVRs [12]. Moon et al. [13] revealed that in patients with and without pre-treatment cirrhosis who had received DAA therapy, DAA-induced SVRs were associated with a lower risk of the occurrence of gastroesophageal variceal bleeding during long-term follow-up; this finding reveals a benefit of the use of DAA therapy. In contrast, in one study, clinically significant PH (HVPG ≥ 10 mmHg) was detected in patients with LC even after the patients had achieved SVRs [14], and in another multicenter prospective study of patients with HCV-related cirrhosis, it was shown that most patients who had achieved SVRs had esophageal varices (EVs) (75%) and persisting clinically significant PH [15]. Furthermore, recent studies have demonstrated that patients with LC develop GEVs after the achievement of DAA-induced SVRs [16,17]. Thus, it remains controversial whether changes related to the progression of GEVs occur in patients with LC.

In this study, we assessed whether DAA-induced HCV eradication reduced the risk of GEV development during follow-up in patients with HCV-related LC. We also investigated other characteristics associated with the progression of GEVs.

## 2. Materials and Methods

### 2.1. Patients

**Figure 1:**
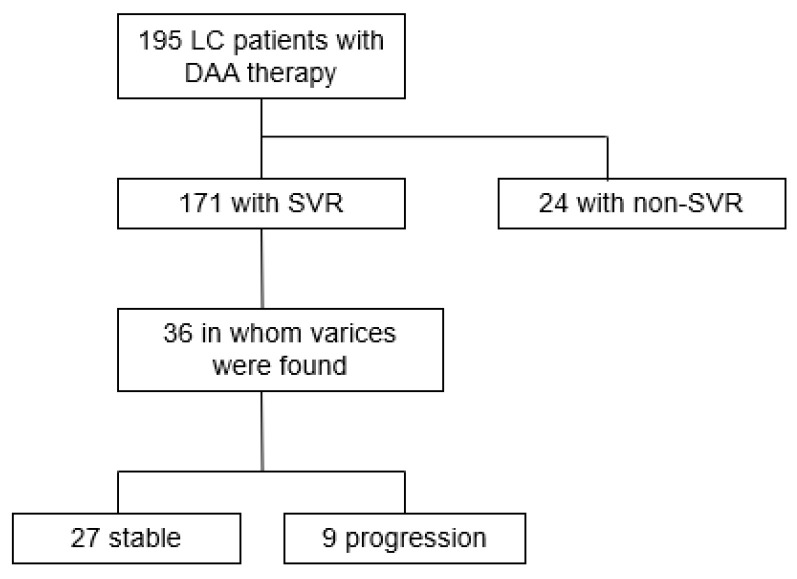
Flow diagram depicting characteristics of patients with liver cirrhosis included in the study. DAA, direct-acting antiviral agent; LC, liver cirrhosis; SVR, sustained virological response.

Clinical and therapeutic data were obtained from the medical records of 195 patients with HCV-related LC who received DAA therapy at our hospital between November 2014 and October 2018 (Figure 1). Out of a total of 171 patients with an SVR, we found that 36 patients had either EVs or gastric varices (GVs) before and after the provision of DAA therapy and were therefore included in this study. Follow-up examinations for a patient were stopped if GEVs in the patient had been treated; if the patient had HCC with major portal vein tumor thrombosis of grade ≥ Vp3; if the patient died; or if the patient was no longer seen as an outpatient. The mean follow-up time was 731.4 days (day 105–1617). This study was performed in accordance with the ethical principles stated in the Declaration of Helsinki (as revised in Brazil in 2013) and was approved by the relevant ethics committee. Informed consent was obtained in the form of opt-out. Those who rejected consent were excluded from the study.

### 2.2. DAA Therapy

The patients received DAA therapy through a combination of daclatasvir and asunaprevir; sofosbuvir; a combination of ledipasvir and sofosbuvir; a combination of ombitasvir, paritaprevir, and ritonavir; or a combination of glecaprevir and pibrentasvir. Levels of HCV ribonucleic acid (RNA) were evaluated using the TaqMan™ test. A patient was considered to have achieved an SVR if no HCV RNA was detected in the patient’s serum after 24 weeks of completion of DAA therapy.

### 2.3. Evaluation of GEVs

Esophagogastroduodenoscopy (EGDS) was performed ≤1 year before the initiation of DAA therapy and every 6 months after the initiation of DAA therapy for all 36 patients included in this study; EGDS was conducted by experienced endoscopists with more than 10 years of experience, and GEVs were evaluated by experienced endoscopists with more than 12 years of experience using photographic records that did not contain patient information. GEVs were assessed using EGDS according to the general rules in Japan for the recording of endoscopic findings associated with GEVs [18]. GEVs were described based on their form (F0: lesion without varicose appearance; F1: straight, small-caliber varices; F2: moderately enlarged, beady varices; and F3: markedly enlarged, nodular, or tumor-shaped varices), and the presence of the red-colored (RC) marks and spots (RC0: absent; RC1: small in number and localized; RC2: intermediate between RC1 and RC3; and RC3: large in number and circumferential). GEVs were graded based on their form and the presence of RC marks and spots as follows: grade 0, no varices; grade 1 (mild GEVs), F1RC0 GEVs; grade 2 (moderate GEVs), F1RC1–3 or F2RC0 GEVs; and grade 3 (severe GEVs), F2RC1–3, F3RC0, or F3RC1–3 GEVs. This grading system has been described previously [19].

Improved GEVs were defined as GEVs in which the grade for form (F0/F1/F2/F3) decreased by one or more and GEVs in which the RC marks and spots that were previously present were absent. A patient was considered to have progressive GEVs if he or she developed new GEVs, if the grade for GEV forms increased by one or more, or if an RC sign appeared in GEVs. Stable GEVs were defined as GEVs that neither improved nor worsened.

### 2.4. Statistical Analysis

Statistical analyses were performed using JMP^®^ Pro 16.0 (SAS Institute; Cary, NC, USA) software. Quantitative variables were compared using Student’s t-test, Mann–Whitney U test, analysis of variance, Kruskal–Wallis test, paired t-test, or Wilcoxon signed-rank test. Categorical variables were compared using Fisher’s exact test or Pearson’s chi-squared test. The Kaplan–Meier method was used to evaluate progression-free periods (with respect to the progression of GEVs), and curves were compared using log-rank tests. Statistical significance was set at *p* < 0.05.

## 3. Results

### 3.1. Characteristics of Patients

**Figure 2:**
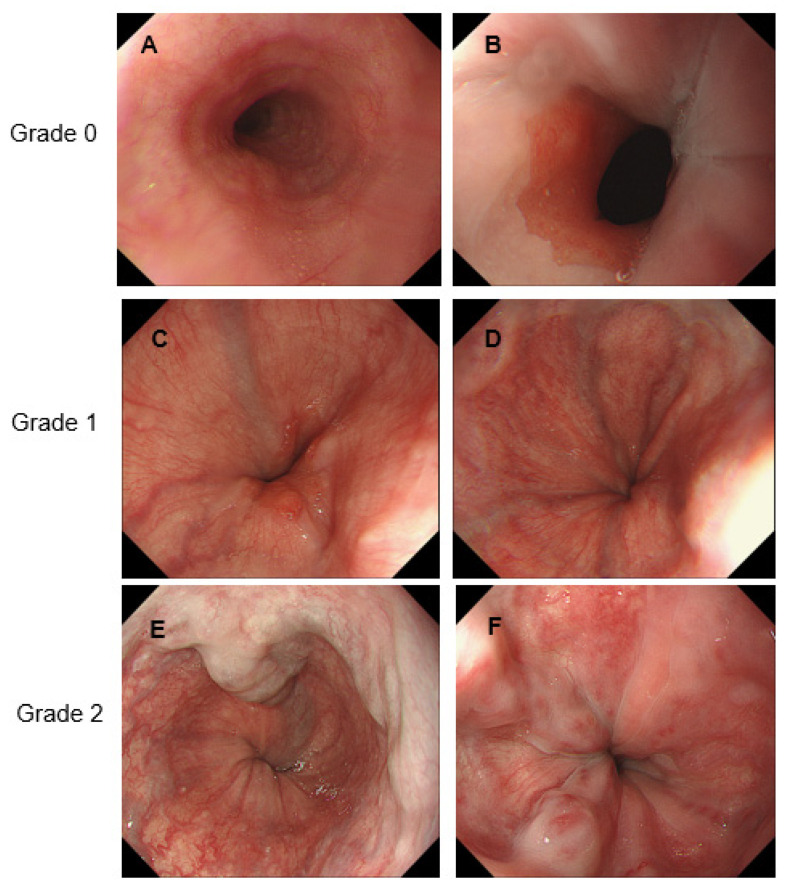
Representative endoscopic images of gastroesophageal varices. (**A**): Normal; (**B**): Normal, (**C**): F1RC0, (**D**): F1RC0, (**E**): F2RC0, (**F**): F2RC1.

Table 1 shows the characteristics of the patients with LC prior to administration of DAA therapy. Patients included 16 men and 20 women, with a median age of 72 years (range: 56–83 years). DAA therapy was administered as follows: a combination of daclatasvir and asunaprevir was administered to 16 patients; sofosbuvir was administered to 11 patients; a combination of ledipasvir and sofosbuvir was administered to 5 patients; a combination of ombitasvir, paritaprevir, and ritonavir was administered to 1 patient; and a combination of glecaprevir and pibrentasvir was administered to 3 patients. Eight patients had a history of being treated for GEVs to prevent the rupture of GEVs. Six of these patients had undergone endoscopic variceal ligation, one endoscopic injection sclerotherapy, and one balloon-occluded antegrade transvenous obliteration. In these patients, endoscopic findings before DAA treatment did not indicate GEVs that required preventive treatment for GEVs rupture. Twenty patients had a history of being treated for HCC before they received DAA therapy. With respect to the endoscopic findings associated with GEVs, before the administration of DAA therapy, 16 patients had only EVs, 2 patients had only GVs, and 17 patients had both EVs and GVs. Only one patient had no GEVs before receiving DAA therapy. Furthermore, with respect to the form of GEVs, 31 patients had F1 GEVs, 4 patients had F2 GEVs, and no patient had F3 GEVs. Regarding the RC marks and spots, 26 patients had RC0 GEVs, 8 patients had RC1 GEVs, and 1 patient had RC2 GEVs; no patient had RC3 GEVs. With respect to GEV grades, which were determined based on the form of GEVs and the presence of RC marks and spots, 1 patient had grade-0 GEVs, 24 patients had grade-1 GEVs, and 11 patients had grade-2 GEVs. None of the patients had grade-3 disease. Endoscopic images of GEV grade 0–2 were shown in Figure 2.

### 3.2. Efficacy of DAA Therapy with Respect to Liver Function and GEVs

By analyzing laboratory data, we evaluated changes between before DAA therapy and at the most recent EGDS after DAA therapy in all 36 patients. (Table 2). We found that there were significant improvements in aspartate transaminase and alanine transaminase levels, platelet counts, serum albumin levels, prothrombin time, and albumin-bilirubin scores, which indicated that DAA therapy caused an improvement in liver function. Additionally, there were significant improvements in type IV collagen levels and the fibrosis-4 index, which are markers of the severity of fibrosis. However, no improvement in GEVs was observed. One patient developed new GEVs, and the number of patients with F2 GEVs increased from 4 to 10. In addition, the number of patients with RC2 GEVs increased from one to four. Therefore, GEV grades tended to worsen after the administration of DAA therapy, although there was no significant difference in GEVs before and after the administration of DAA therapy (*p* = 0.098).

### 3.3. Differences in Pre-Treatment Characteristics of Patients Who Had Stable GEVs or Progressive GEVs after Receiving DAA Therapy

After the patients had been treated with DAAs, 27 patients had stable GEVs, and 9 patients had progressive GEVs; there were no improvements in GEVs. We compared the pre-treatment characteristics of patients with stable and progressive GEVs (Table 3). There were no significant differences with respect to age, sex, medical history, past history of HCC treatment, liver function, or fibrosis. However, regarding GEV grades, there was a significant difference between the two groups; compared to the patients with stable GEV, the patients with progressive GEVs included a greater proportion of those who had grade-2 GEVs before receiving DAA therapy (*p* = 0.027).

**Figure 3:**
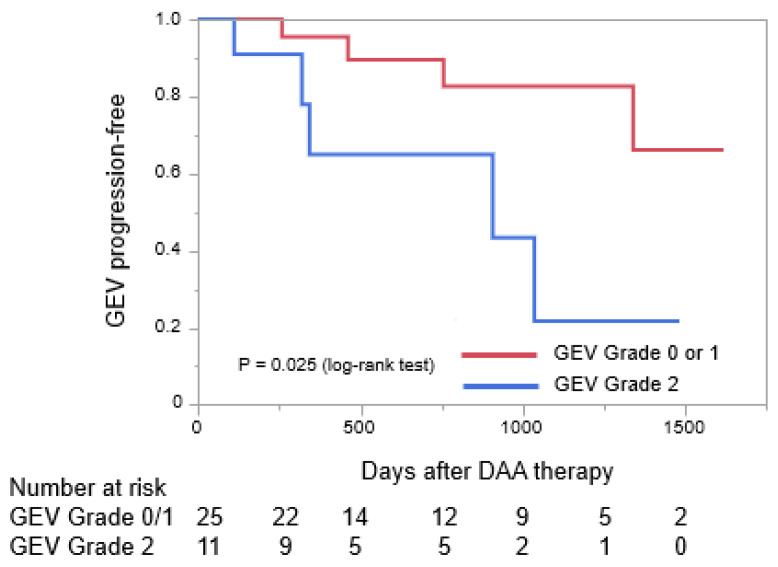
Curves for periods without the progression of gastroesophageal varices were created using the Kaplan–Meier method and compared using the log-rank test. DAA, direct-acting antiviral agent; GEV, gastroesophageal varix.

We also investigated the patient risk factors that contributed to the progression of GEVs (Table 3) and found that there were no significant risk factors associated with age, sex, medical history, past history of HCC treatment, and liver function. Although we discovered that the presence of both EVs and GVs, GEVs with forms of grade F2 or higher and GEVs with the RC marks and spots of grades RC1–2 were not significant risk factors, the odds ratio for GEV grades ≥ 2 was 5.83 (*p* = 0.04). This indicates that having GEVs of grade-2 or higher was a risk factor for the worsening of GEVs after receiving DAA therapy. We then compared the GEV-progression-free period after receiving DAA therapy in the patients with GEVs of grades 0–1 to that in patients with GEVs of grade-2. As shown in Figure 3, patients with GEVs of grade-2 had significantly shorter progression-free periods than patients with GEVs of grades 0–1 (*p* = 0.025).

## 4. Discussion

The use of DAAs for the treatment of HCV infections has led to an improvement in the proportion of patients who achieve SVRs (>95%); DAA therapy is associated with short administration periods and few side effects [20]. It has been reported that patients with LC who achieve SVRs with DAA therapy show improvements in liver function and fibrosis, with improvements in aspartate transaminase levels, alanine transaminase levels, serum albumin levels, type IV collagen levels, and the fibrosis-4 index [21,22]. Furthermore, in certain studies in which liver function in patients with decompensated cirrhosis was evaluated, it was found that the achievement of DAA-induced SVRs was associated with improvements in the scores according to the model for end-stage liver disease and a decreased occurrence of decompensation events [23,24,25]. In the present study, we found that among the included patients, there was a significant improvement in liver function after the administration of DAA therapy. Therefore, the administration of DAA therapy to patients with LC provides some benefits associated with liver function and fibrosis.

Recently, an increasing number of studies and cases regarding PH and GEVs have been reported. However, it remains controversial whether the achievement of DAA-induced SVRs leads to improvements in PH and GEVs. In our study, because we observed that the patient’s liver function improved after the administration of DAA therapy, we expected that there would be improvements in the condition of GEVs; however, no improvements were observed in any patient. In contrast, we found that 9 of the 36 patients had progressive GEVs, which was an unexpected finding. As mentioned previously, it was shown that among patients with and without pre-treatment cirrhosis who had received DAA therapy, DAA-induced SVRs were associated with a lowered risk of the occurrence of gastroesophageal variceal bleeding [13,26,27]. This was supported by the finding that HCV eradication was associated with the reversal of liver fibrosis [23,28,29] and improvements in PH [15,29]. Interestingly, Moon et al. [13] reported that although DAA-induced SVRs reduced the risk of variceal bleeding, 4.8% of patients with cirrhosis developed variceal bleeding despite the achievement of SVRs. Puigvehí et al. [17] found that patients who did not have GEVs before they received DAA therapy had a very low risk of having progressive GEVs; in contrast, patients who had GEVs before they received DAA therapy had a high risk of having progressive GEVs, even if they had achieved SVRs after receiving DAA therapy. Puigvehí et al. likewise demonstrated that GEV progression occurred in 13.2% of patients who had achieved SVRs. Patients who had GEVs before receiving antiviral treatment were found to have a high risk of developing GEVs during follow-up periods (24% of patients with pre-treatment GEVs developed GEVs during follow-up periods, and 1.6% of patients without pre-treatment GEVs developed GEVs during follow-up periods). Thus, it was suggested that with the administration of DAA therapy, GEVs may worsen in some patients with LC, even after the patients have achieved DAA-induced SVRs.

Through a multivariate analysis, Yuri et al. [16] revealed that patients who had GEVs with forms of grade F2 or higher had a high risk of developing GEV progression; in this study, with respect to GEV progression, a significant difference was identified between patients with no EVs or F1 EVs and patients with F2 or F3 EVs (*p* = 0.0003). Furthermore, Mandorfer et al. [12] found that regarding the effect of SVRs achieved in response to interferon-free therapies, compared with patients with Child–Pugh stage-A cirrhosis, patients with Child–Pugh stage-B cirrhosis were less likely to have a decreased HVPG after receiving treatment (hazard ratio: 0.103, *p* = 0.006). Herein, we showed that the presence of grade-2 GEVs, including F2 GEVs or RC+ GEVs, was a risk factor for developing GEV progression after receiving DAA therapy. Additionally, after receiving DAA therapy, grade-2 GEVs began to worsen considerably earlier than GEVs of grades-0 and 1. Therefore, compared to liver function and fibrosis, GEVs may be less likely to improve after HCV eradication in patients with LC. According to a report by Libânio et al. [30], it is assumed that there is a point at which liver damage is so advanced that it can no longer be reversed. A growing body of evidence suggests that there is a point of no return after which the state of GEVs cannot be improved, even after HCV eradication. However, DAA-induced SVRs significantly reduce the risk of variceal bleeding in patients without cirrhosis or pre-treatment varices [13]. Prompt treatment of HCV infection before the development of cirrhosis significantly decreases the risk of GEV progression.

This study had certain limitations. First, this was a single-center, retrospective observational study, and a small number of patients was analyzed. By conducting prospective and multicenter studies, we will be able to clarify the long-term outcomes of GEVs in patients with LC treated using DAAs. Second, as none of the patients included in this study had grade-3 GEVs, these could not be evaluated. However, we believe that results for grade-3 GEVs would be similar to those for grade-2 GEVs. Furthermore, 20 patients had a history of HCC before DAA therapy and there was no development of HCC during DAA therapy. However, 14 patients including 9 in stable GEVs (9/27, 33.3%) and 5 in progressive GEVs (5/9, 55.5%) developed HCC after DAA therapy. Although the development of HCC was not a risk factor in progressive GEV (Odd ratio 2.50, *p*-value 0.24), HCC progression and treatment may have affected the course of GEV. Future studies will be conducted separately for GEVs with and without HCC.

## 5. Conclusions

The findings of this study showed that liver function and fibrosis improved with DAA therapy; however, no improvement in GEVs was observed. Furthermore, grade-2 GEVs were found to have the potential to worsen after receiving DAA therapy. Therefore, it is recommended that patients with GEVs undergo careful endoscopic surveillance after receiving DAA therapy. We also recommend that DAA therapy for HCV infection be administered before the development of LC.

## Figures and Tables

**Table 1:** Characteristics of patients.

*n*	36
Male/female	16/20
Age (years), median (range)	72 (56–83)
Dacratasvir-asunaprevir/sofosbuvir/ledipasvir-sofosbuvir/ombitasvir-paritaprevir-ritonavir/glecaprevir-pibrentasvir	16/11/5/1/3
Child–Pugh cirrhosis classification, A/B/C	30/6/0
Past history of GEV treatment, yes/no	8/28
Past history of HCC treatment, yes/no	20/16
Presence of GEVs before DAA, yes/no	35/1
GEV, EV/GV/EV + GV	16/2/17
Form, F1/F2/F3	31/4/0
RC sign, RC0/RC1/RC2/RC3	26/8/1/0
GEV grade, 0/1/2/3	1/24/11/0

DAA, direct-acting antiviral agents; EV, esophageal varix; GEV, gastroesophageal varix; GV, gastric varix; HCC, hepatocellular carcinoma; RC, red-colored.

**Table 2:** Comparison of liver function and GEVs before and after DAA therapy.

	Before DAAs	After DAAs	*p*-Value
AST (IU/L)	67.4 [46.9]	30.5 [9.7]	<0.001
ALT (IU/L)	59.8 [36.3]	20.6 [9.3]	<0.001
Platelet count (×10^4^/mm^3^)	8.2 [3.0]	9.6 [3.8]	0.002
Serum albumin (g/dL)	3.45 [0.37]	3.99 [0.63]	<0.001
Total bilirubin (mg/dL)	0.95 [0.37]	1.04 [0.48]	0.14
Prothrombin time (%)	77.5 [16.0]	86.4 [15.1]	0.002
ALBI	2.15 [0.35]	2.58 [0.53]	<0.001
Type IV collagen	258 [75.1]	210 [66.4]	<0.001
FIB-4 index	9.29 [4.69]	6.27 [3.00]	<0.001
Child–Pugh, A/B/C	30/6/0	35/1/0	0.14
GEV, none/EV/GV/EV + GV	1/16/2/17	0/17/2/17	0.79
Form, none/F1/F2	1/31/4	0/26/10	0.13
RC sign, RC0/RC1/RC2	27/8/1	25/7/4	0.38
GEV grade, 0/1/2/3	1/24/11/0	0/22/9/5	0.098

Data are presented as the mean [standard deviation] or *n*. The mean follow-up time after DAAs was 731.4 days (day 105–day 1617). ALBI, albumin-bilirubin; ALT, alanine transaminase; AST, aspartate transaminase; DAA, direct-acting antiviral agent; EV, esophageal varix; FIB-4 index, fibrosis 4 index; GEV, gastroesophageal varix; GV, gastric varix; RC, red-colored.

**Table 3:** Comparison of stable GEVs and progressive GEVs and factors contributing to progressive GEV before DAA therapy.

	Stable GEVs	Progressive GEVs	Odds Ratio	*p*-Value	95% Confidence Interval
*n*	27	9			
Male/Female	11/16	5/4	1.82	0.44	0.39–8.33
Age, years (range)	72 (56–83)	72 (59–83)	1.02	0.67	0.91–1.13
dacratasvir-asunaprevir/sofosbuvir/ledipasvir-sofosbuvir/ombitasvir-paritaprevir-ritonavir/glecaprevir-pibrentasvir	12/9/2/1/3	4/2/3/0/0		0.20	
Child–Pugh; A/B/C	23/4/0	7/2/0	1.64	0.61	0.25–10.95
Past history of GEVs treatment, yes/no	6/21	2/7	1.00	1.00	0.16–6.14
Past history of HCC treatment, yes/no	15/12	5/4	1.00	1.00	0.22–4.56
Presence of GEV varices before DAA, yes/no	27/0	8/1	0.00	0.08	0.00–1.26
AST, mean [SD]	65.2 [35.3]	73.8 [74.1]	2.57	0.64	0.05–126.2
ALT, mean [SD]	48.8 [21.2]	52.4 [50.8]	1.57	0.80	0.05–45.7
Platelet count, mean ×10^4^/mm^3^ [SD]	8.3 [3.1]	7.8 [2.6]	0.51	0.68	0.02–12.46
Serum albumin, mean g/dL [SD]	3.44 [0.3]	3.49 [0.5]	1.78	0.74	0.06–51.52
Total bilirubin, mean mg/dL [SD]	0.91 [0.3]	1.07 [0.4]	4.79	0.28	0.29–80.06
Prothrombin time, mean % [SD]	78.3 [15.9]	74.9 [17.0]	0.40	0.57	0.02–9.57
ALBI, mean [SD]	−2.2 [0.3]	−2.1 [0.5]	0.90	0.95	0.03–22.92
Type IV collagen, mean [SD]	249.0 [80.6]	281.5 [55.9]	5.33	0.29	0.23–125.14
FIB-4 index, mean [SD]	8.9 [4.1]	10.4 [6.2]	3.87	0.41	0.15–96.02
GEV; None/EV/GV/EV + GV	0/14/2/11	1/2/0/6		0.10	
EV + GV			3.82	0.14	0.64–22.7
Form; None/F1/F2	0/24/3	1/7/1		0.10	
F2			1.14	0.91	0.10–12.8
RC sign; RC0/RC1/RC2	22/5/0/	4/3/1		0.13	
RC1-2			2.64	0.27	0.47–14.9
GEV grade; 0/1/2	0/21/6	1/3/5		0.027	
Grade-2			5.83	0.04	1.07–31.76

Data are presented as the median (range), mean [standard deviation], or *n*. ALBI, albumin-bilirubin; ALT, alanine transaminase; AST, aspartate transaminase; DAA, direct-acting antiviral agent; EV, esophageal varix; FIB-4 index, fibrosis 4 index; GEV, gastroesophageal varix; GV, gastric varix; HCC, hepatocellular carcinoma; RC, red-colored.

## Data Availability

Data available on request due to privacy. The data presented in this study are available on request from the corresponding author. The data are not publicly available due to their containing information that could compromise the privacy of research participants.
